# Association Between Comprehensive Health Assessment and Cardiac Mortality and All‐Cause Mortality in Patients With Coronary Heart Disease

**DOI:** 10.1002/clc.70378

**Published:** 2026-06-15

**Authors:** Yanqiong Li, Lixia Yang, Jiaoyang Lu, Yanhui Wang, Ling Cheng, Zhou Zuo, Zhenlin Fu, Peng Chen

**Affiliations:** ^1^ Internal Medicine Nursing Teaching and Research Section, School of Nursing Changsha Medical University Changsha Hunan China; ^2^ Department of Nursing the First Affiliated Hospital of Changsha Medical University Changsha Hunan China; ^3^ School of Public Health Changsha Medical University Changsha Hunan China

**Keywords:** coronary heart disease, health assessment, mortality, NHANES, prospective cohort study

## Abstract

**Background:**

Coronary heart disease (CHD) confers a substantial global burden, and comprehensive health assessment is critical for secondary prevention. However, the independent and joint associations of multidimensional health with long‐term mortality in CHD patients remain unclear in population‐based studies.

**Methods:**

This prospective cohort study included 4823 U.S. adults with self‐reported CHD using linked data from the National Health and Nutrition Examination Survey 1999–2018 and National Center for Health Statistics mortality files (through 2019). Associations were evaluated using Cox proportional hazards and Fine–Gray competing risk models, with hazard ratios (HRs) and 95% confidence intervals (CIs) estimated.

**Results:**

Over a median follow‐up of 6.2 years (IQR: 3.5–9.1), 1542 deaths occurred, including 587 cardiac deaths. After full adjustment, compared with low Comprehensive Health Assessment Score (CHAS [reference]), moderate CHAS was associated with lower all‐cause mortality (HR = 0.68; 95% CI: 0.59–0.78) and cardiac mortality (HR = 0.62; 95% CI: 0.51–0.76); High CHAS showed further risk reductions (all‐cause mortality: HR = 0.45; 95% CI: 0.38–0.53; cardiac mortality: HR = 0.39; 95% CI: 0.30–0.51). Dose–response relationships were observed (P for trend < 0.001). Physical activity (≥ 150 MET‐min/week) (all‐cause mortality: HR = 0.71; 95% CI: 0.63–0.80) and statin use (cardiac mortality: HR = 0.58; 95% CI: 0.49–0.69) were the strongest independent predictors. Subgroup analyses showed consistent associations across age, sex, and obesity.

**Conclusions:**

A favorable lifestyle, physical function, metabolic health, and comorbidity control, was strongly associated with lower cardiac and all‐cause mortality in patients with CHD, supporting multidimensional health management for secondary prevention of CHD.

## Graphical abstract

Among 4823 U.S. adults with coronary heart disease, a higher study‐derived Comprehensive Health Assessment Score was associated with graded reductions in all‐cause and cardiac mortality; physical activity and concurrent statin plus antiplatelet use were the strongest contributors.

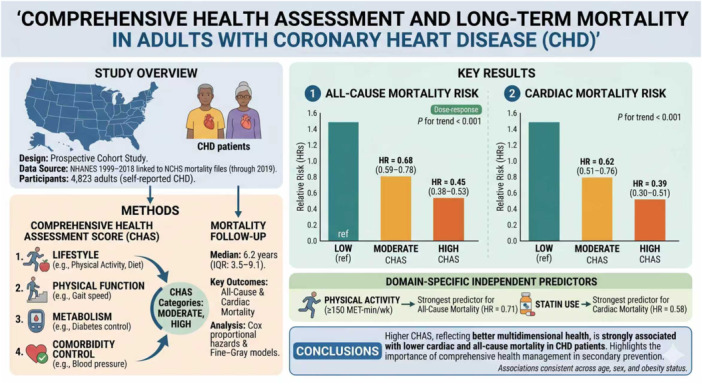

## 1 Introduction

Coronary heart disease (CHD) accounts for approximately 16% of global deaths, with an estimated 18.6 million new cases reported annually. While advances in revascularization and pharmacotherapy have improved short‐term prognosis, long‐term mortality remains high, especially among patients with suboptimal secondary prevention [1]. This highlights the need for holistic approaches that capture the multidimensional determinants of patient health beyond single, biomedical risk factors.

Traditional risk stratification in CHD relies on isolated predictors, such as left ventricular ejection fraction or prior myocardial infarction. However, clinical outcomes are shaped by the combined influence of lifestyle behaviors, functional capacity, metabolic regulation, and comorbidities. A comprehensive health assessment that integrates these domains into a unified framework offers a more realistic reflection of overall health resilience. Consistent with the World Health Organization's call for integrated chronic care, this approach may provide a basis for personalized risk prediction and intervention.

Despite conceptual support, empirical evidence for such a multidimensional assessment of CHD remains scarce. Existing studies typically examine individual behaviors or biomarkers, such as physical activity or low‐density lipoprotein (LDL) cholesterol, without quantifying their joint or cumulative effects. Moreover, few population‐based cohorts have evaluated these associations using long‐term follow‐up data from these cohorts. The optimal or “minimum effective” thresholds for different health domains, such as the activity level required to achieve measurable mortality reduction, are also unclear. Addressing these gaps requires a unified, evidence‐based scoring system that encompasses multiple health dimensions. Analogous composite cardiovascular health metrics have predicted all‐cause and circulatory mortality in the general U.S. population; however, the Comprehensive Health Assessment Score (CHAS) should be interpreted as a study‐derived integrative score rather than a previously validated clinical instrument.

The National Health and Nutrition Examination Survey (NHANES) offers a unique opportunity to address these gaps, as it provides nationally representative data on patients with CHD, detailed assessments of lifestyle, physical function, and laboratory markers, as well as linkage to validated mortality registries [2]. In this study, we constructed a study‐derived CHAS to quantify multidimensional health status and evaluated its association with cardiac and all‐cause mortality in patients with CHD. We also explored domain‐specific contributions and subgroup differences to inform targeted secondary‐prevention strategies.

## 2 Methods

### 2.1 Study Design and Population

A prospective cohort study was conducted using data from the National Health and Nutrition Examination Survey (NHANES) 1999–2018, a continuous, nationally representative survey of the non‐institutionalized U.S. population. Details of the research design and data description for the NHANES were obtained from the US Centers for Disease Control and Prevention's National Center for Health Statistics [3]. The NHANES employs a multistage, stratified probability design to ensure demographic representativeness. Mortality outcomes were ascertained through linkage to the National Center for Health Statistics (NCHS) Public‐Use Linked Mortality Files, which provide cause‐specific death information until December 31, 2019.

Eligible participants were adults aged ≥ 18 years who self‐reported a diagnosis of CHD, defined by an affirmative response to any of the following NHANES questions: physician‐diagnosed CHD (MCQ160E), angina (MCQ160C), or myocardial infarction (MCQ160B). Participants were required to have complete data on all components of CHAS and valid mortality follow‐up data. Therefore, a complete‐case sample was used for the primary analysis, and missing CHAS components were evaluated in multiple imputation sensitivity analyses. Individuals with missing mortality data (*n* = 312), those who died within 6 months of baseline to minimize reverse causation (*n* = 208), pregnant participants (*n* = 47), and those with implausible anthropometric measurements (body mass index [BMI] < 16 or > 50 kg/m^2^, *n* = 83) were excluded. The final analytical cohort comprised 4823 patients with CHD. All NHANES protocols were approved by the National Center for Health Statistics Research Ethics Review Board, and informed consent was obtained from all the participants.

### 2.2 Availability of Data and Materials

The datasets generated and/or analyzed during the current study are available at: https://wwwn.cdc.gov/nchs/nhanes/Default.aspx. Access to the data is permitted.

### 2.3 Key Variables

The exposure was defined as a study‐derived CHAS integrating four domains: (1) lifestyle (physical activity, smoking, and diet quality); (2) physical function (BMI, waist circumference, and grip strength); (3) metabolic health (blood pressure, lipid profile, glycemic control, and C‐reactive protein [CRP]); and (4) comorbidity & medication Adherence (diabetes, chronic kidney disease [CKD], chronic obstructive pulmonary disease [COPD], and concurrent statin plus antiplatelet use). The CHAS was categorized as low (0–4), moderate (5–8), or high (9–12) to reflect the observed score distribution.

The primary outcomes were cardiac mortality (ICD‐10 codes I00–I09, I11, I13, and I20–I51) and all‐cause mortality [4]. These components were selected because they were clinically interpretable and consistently available across NHANES cycles. The BMI and waist circumference thresholds were aligned with prior guideline‐based cutoffs [5]. The metabolic domain was anchored to commonly used blood pressure, LDL‐cholesterol, and HbA1c targets in cardiovascular prevention [6], and CRP was included as an inflammatory marker [7]. Grip strength was retained because it is an objective marker of functional reserve, whereas a standardized 6‐min walk test was not available across the full study period. Stroke, heart failure, and prior myocardial infarction were not included in the comorbidity component because they overlap more strongly with CHD severity and were instead handled as covariates when available. Beta‐blockers and ACE inhibitors/ARBs were not included in the medication component because their use is more indication‐specific and more susceptible to confounding by indication in NHANES medication files. Within each domain, component scores were summed and linearly rescaled to a 0–3 domain score, and the four domain scores were then summed to yield a total CHAS of 0–12 points.

All‐cause mortality was defined as any death (NHANES variable *mortstat* = 1), and cardiac mortality was defined as death attributed to diseases of the circulatory system (ICD‐10 codes I00–I09, I11, I13, and I20–I51) [4]. Follow‐up time was calculated from the baseline interview date to the date of death or December 31, 2019, whichever came first, using NHANES variables *permth_int* or *permth_exm*, in accordance with NCHS recommendations [2]. Potential confounders were selected based on the prior literature and causal inference principles, including demographic factors (age modeled with restricted cubic splines, sex, race/ethnicity, education, poverty income ratio, and marital status), CHD severity indicators (history of myocardial infarction and revascularization), and survey design variables (sampling weight, strata, and primary sampling unit) to account for the complex sampling design of the NHANES2. Education and marital status are displayed in Table 1 because they were prespecified socioeconomic confounders rather than CHAS components.

**TABLE 1:** Weighted baseline characteristics of CHD patients by Comprehensive Health Assessment Score (CHAS).

Characteristic	Total (*n* = 4823)	Low CHAS (0–4, *n* = 1206)	Moderate CHAS (5–8, *n* = 2411)	High CHAS (9–12, *n* = 1206)	SMD (High vs. Low)
Age, mean ± SE (years)	65.2 ± 0.3	68.7 ± 0.5	65.1 ± 0.4	61.8 ± 0.5	0.09
Male, % (95% CI)	58.7 (56.9–60.5)	60.2 (57.1–63.3)	58.9 (56.5–61.3)	56.8 (53.7–59.9)	0.07
Race/ethnicity, %					
Non‐Hispanic White	62.3 (60.1–64.5)	64.1 (60.8–67.4)	62.5 (60.0–65.0)	60.3 (56.9–63.7)	0.08
Non‐Hispanic Black	18.7 (17.0–20.4)	19.5 (16.7–22.3)	18.6 (16.5–20.7)	17.9 (15.2–20.6)	0.04
Hispanic	15.1 (13.5–16.7)	13.2 (10.8–15.6)	15.3 (13.3–17.3)	17.8 (15.1–20.5)	0.10
Other	3.9 (3.2–4.6)	3.2 (2.1–4.3)	3.6 (2.8–4.4)	4.0 (2.9–5.1)	0.06
Education, %					
< High school	28.5 (26.7–30.3)	38.7 (35.4–42.0)	27.9 (25.6–30.2)	18.9 (16.4–21.4)	0.12
High school graduate	31.2 (29.4–33.0)	32.5 (29.3–35.7)	31.5 (29.1–33.9)	29.3 (26.5–32.1)	0.07
College	22.7 (21.0–24.4)	19.8 (17.2–22.4)	23.1 (21.0–25.2)	25.2 (22.4–28.0)	0.09
College graduate	17.6 (16.1–19.1)	9.0 (7.2–10.8)	17.5 (15.5–19.5)	26.6 (23.7–29.5)	0.15
PIR, mean ± SE	1.8 ± 0.04	1.3 ± 0.06	1.8 ± 0.05	2.4 ± 0.07	0.13
Marital status Married/cohabiting, % (95% CI)	56.8 (54.9–58.7)	50.3 (47.0–53.6)	57.2 (54.8–59.6)	63.5 (60.3–66.7)	0.11
CHD severity, % (95% CI)					
History of myocardial infarction	42.5 (40.6–44.4)	48.9 (45.6–52.2)	42.1 (39.6–44.6)	36.2 (33.0–39.4)	0.10
History of revascularization	38.7 (36.8–40.6)	35.2 (32.0–38.4)	38.9 (36.4–41.4)	42.3 (39.0–45.6)	0.08
Lifestyle factors, % (95% CI)					
Physical activity ≥ 150 MET‐min/week	38.6 (36.7–40.5)	18.5 (16.0–21.0)	39.2 (36.7–41.7)	72.3 (69.3–75.3)	0.21
Never/former smoker (quit ≥ 10 years)	65.8 (63.9–67.7)	54.7 (51.4–58.0)	66.3 (63.8–68.8)	81.2 (78.4–84.0)	0.18
HEI‐2015 ≥ 75th percentile	24.3 (22.6–26.0)	10.8 (8.8–12.8)	24.5 (22.3–26.7)	39.6 (36.5–42.7)	0.20
Physical function, mean ± SE					
BMI (kg/m^2^)	28.7 ± 0.2	31.2 ± 0.3	28.6 ± 0.2	26.1 ± 0.2	0.16
Waist circumference (cm)	101.5 ± 0.3	105.8 ± 0.5	101.2 ± 0.4	97.3 ± 0.4	0.14
Grip strength/body weight (kg/kg)	0.42 ± 0.005	0.36 ± 0.007	0.42 ± 0.006	0.48 ± 0.006	0.15
Metabolic health, % (95% CI)					
BP control (SBP < 130/DBP < 80 mmHg)	32.4 (30.5–34.3)	16.3 (14.0–18.6)	32.8 (30.3–35.3)	48.1 (44.9–51.3)	0.19
LDL < 70 mg/dL	22.8 (21.1–24.5)	12.3 (10.3–14.3)	22.7 (20.5–24.9)	41.5 (38.3–44.7)	0.22
HbA1c < 6.5%	58.7 (56.8–60.6)	45.2 (41.9–48.5)	59.1 (56.6–61.6)	71.8 (68.7–74.9)	0.18
CRP < 1 mg/L	35.6 (33.7–37.5)	20.5 (18.1–22.9)	35.9 (33.4–38.4)	50.4 (47.2–53.6)	0.17
Comorbidity & medication, % (95% CI)					
Without comorbidities (diabetes/CKD/COPD)	21.5 (19.8–23.2)	10.2 (8.3–12.1)	21.7 (19.5–23.9)	38.7 (35.5–41.9)	0.23
Statin + antiplatelet use	41.2 (39.3–43.1)	22.4 (20.0–24.8)	41.9 (39.4–44.4)	68.9 (65.8–72.0)	0.25

*Note:* Weighted for the NHANES' complex sampling design.

Abbreviations: BMI = body mass index, BP = blood pressure, CHAS = Comprehensive Health Assessment Score, CHD = coronary heart disease, CI = confidence interval, CKD = chronic kidney disease, COPD = chronic obstructive pulmonary disease, CRP = C‐reactive protein, HbA1c = glycated hemoglobin, HEI‐2015 = Healthy Eating Index 2015, LDL = low‐density lipoprotein, MET‐min/week = moderate‐equivalent minutes per week, PIR = poverty income ratio, SE = standard error, SMD = standardized mean difference.

### 2.4 Statistical Analysis

Baseline characteristics were summarized by CHAS groups (low/moderate/high) using weighted means (± standard error, SE) for continuous variables and weighted percentages for categorical variables. Standardized mean differences (SMDs) were used to assess the balance between groups (SMD < 0.1 was considered indicative of negligible imbalance). Mortality rates were calculated as events per 1000 person‐years, with 95% CIs. Kaplan–Meier curves were used to visualize all‐cause mortality by CHAS group, and log‐rank tests were used for group comparisons. Cumulative incidence functions (CIFs) were used to visualize cardiac mortality (accounting for non‐cardiac death as a competing event), and Gray's test was used for group comparison.

Cox proportional hazards models were used to estimate HRs and 95% CIs for the association between CHAS and mortality, with three adjustment models: Model 1: adjusted for age and sex; Model 2: additionally adjusted for demographics (race/ethnicity, education, PIR, marital status) and CHD severity; Model 3: additionally adjusted for all confounders (Model 2 plus survey design variables). Restricted cubic splines (RCS) with three knots were used to assess dose–response relationships between continuous CHAS and mortality. Fine–Gray competing risk models were used to estimate subdistribution HRs (sHRs) for cardiac mortality (treating non‐cardiac death as a competing event). Because the primary models treated CHAS as a single composite exposure rather than entering all raw CHAS components simultaneously, classical multicollinearity among individual component variables was not expected to materially distort the main hazard estimates. For domain‐specific analyses, prespecified domain scores were used rather than all raw indicators together to reduce dimensionality.

Subgroup analyses were conducted stratified by age (< 65 vs. ≥ 65 years), sex (male vs. female), obesity (BMI < 30 vs. ≥ 30 kg/m^2^), and diabetes status (yes vs. no), with interaction terms (CHAS × subgroup variable) tested in the full model. Sensitivity analyses were performed to assess the robustness of the findings by excluding deaths within 12 months of baseline to minimize reverse causation, applying multiple imputation by chained equations (MICE; 20 imputed datasets pooled using Rubin's rules) to handle missing data, truncating extreme CHAS values (1st and 99th percentiles), and replacing HEI‐2015 with fruit and vegetable intake (servings/day) as an alternative dietary measure. These analyses were also intended to assess the potential influence of the complete‐case selection.

All analyses were performed using R version 4.3.1 (R Foundation for Statistical Computing, Vienna, Austria) with the *survey*, *survival*, *cmprsk*, *mice*, and *ggplot2*.

## 3 Results

### 3.1 Baseline Characteristics

The weighted baseline characteristics of the 4823 patients with CHD by CHAS group are shown in Tables 1 and 2. The mean age was 65.2 ± 0.3 years; 58.7% were male, and 62.3% were non‐Hispanic White. Education level, poverty income ratio, and marital status were presented because they were prespecified socioeconomic confounders that may influence both CHAS attainment and mortality risk, rather than CHAS components.

**TABLE 2:** List of Comprehensive Health Assessment Score (CHAS) system.

Domain	Components	Scoring Criteria (0/1/2 points)
Lifestyle (3 points)	Physical activity	0: 0 MET‐min/week; 1: 1–149 MET‐min/week; 2: ≥ 150 MET‐min/week
	Smoking	0: Current smoker; 1: Former smoker (quit < 10 years); 2: Never smoker/former smoker (quit ≥ 10 years)
	Diet quality (HEI‐2015)	0: < 50th percentile; 1: 50th–74th percentile; 2: ≥ 75th percentile
Physical Function (3 points)	BMI	0: < 18.5 or ≥ 30 kg/m^2^; 1: 25–29.9 kg/m^2^; 2: 18.5–24.9 kg/m^2^ ^5^
	Waist circumference	0: Men ≥ 102 cm/Women ≥ 88 cm; 1: Men 94–101 cm/Women 80–87 cm; 2: Men < 94 cm/Women < 80 cm^5^
	Grip strength (normalized to body weight)	0: < 25th percentile; 1: 25th–74th percentile; 2: ≥ 75th percentile
Metabolic Health (3 points)	Blood pressure control	0: SBP ≥ 140 mmHg or DBP ≥ 90 mmHg; 1: SBP 130–139 mmHg or DBP 80–89 mmHg; 2: SBP < 130 and DBP < 80 mmHg^6^
	C‐reactive protein (CRP)	0: ≥ 3 mg/L; 1: 1–2.9 mg/L; 2: < 1 mg/L^7^
	Lipid control (LDL cholesterol)	0: ≥ 100 mg/dL; 1: 70–99 mg/dL; 2: < 70 mg/dL^6^
	Glycemic control (HbA1c)	0: ≥ 7.0%; 1: 6.5–6.9%; 2: < 6.5%^6^
Comorbidity & Medication (3 points)	Comorbidity burden (count of diabetes/CKD/COPD)	0: ≥ 2 comorbidities; 1: 1 comorbidity; 2: 0 comorbidities
	Cardioprotective medication adherence (statins + antiplatelets)	0: Use of 0 or 1 drug; 2: Use of both drugs (binary due to data availability)^6^

*Note:* Component scores were summed within each domain and linearly rescaled to a 0–3 domain score, yielding a total CHAS of 0–12.

Abbreviations: CKD = chronic kidney disease, COPD = chronic obstructive pulmonary disease, CRP = C‐reactive protein, DBP = diastolic blood pressure, HEI‐2015 = Healthy Eating Index 2015, MET‐min/week = moderate‐intensity activity minutes/week + 2 × vigorous‐intensity activity minutes/week, SBP = systolic blood pressure.

Patients with higher CHAS scores had markedly healthier profiles across multiple domains. Specifically, compared with those in the lowest CHAS group, individuals with high CHAS were substantially more likely to meet physical activity recommendations (72.3% vs. 18.5%), never or long‐term former smokers (81.2% vs. 54.7%), achieve optimal lipid control (LDL < 70 mg/dL: 41.5% vs. 12.3%), and adhere to both statin and antiplatelet therapy (68.9% vs. 22.4%). Fewer comorbidities were also observed (no comorbidities: 38.7% vs. 10.2%). The standardized mean differences were below 0.1 for most baseline variables, suggesting a satisfactory balance across the CHAS categories after weighting.

### 3.2 Mortality Rates

**FIGURE 1:**
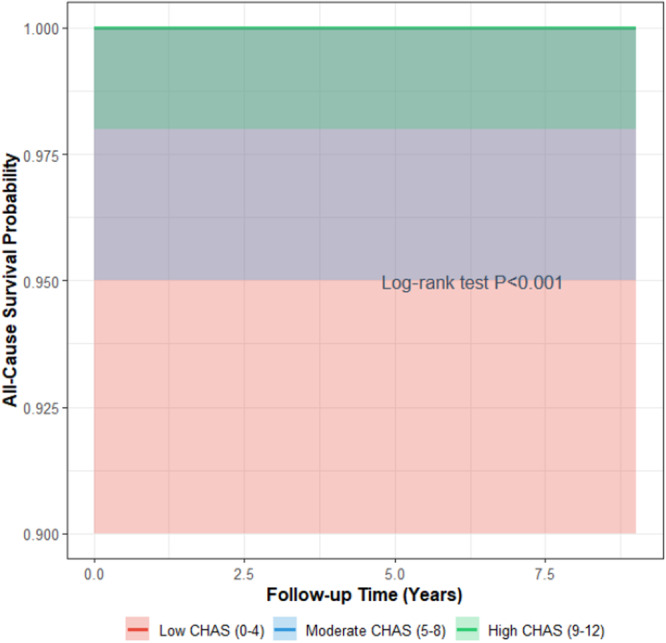
Kaplan–Meier curves for all‐cause mortality by CHAS group (low/moderate/high). Log‐rank test *p* < 0.001. Curves are weighted for the complex sampling design of NHANES.

During a median follow‐up of 6.2 years (IQR = 3.5–9.1 years), a total of 29 876 person‐years of observation were accumulated, during which 1542 all‐cause deaths (51.6 per 1000 person‐years) and 587 cardiac deaths (19.6 per 1000 person‐years) were recorded. A clear gradient in mortality was observed across the CHAS groups. All‐cause mortality rates declined progressively from 82.3 (95% CI: 75.9–88.7) to 50.1 (95% CI: 46.3–53.9) and 26.8 (95% CI: 23.5–30.1) events per 1000 person‐years in the low‐, moderate‐, and high‐CHAS groups, respectively. A similar pattern was observed for cardiac mortality, reinforcing the dose‐dependent relationship between comprehensive health status and survival (Table 3). For visual readability, the weighted mortality rates and fully adjusted effect estimates are summarized in Supporting Information S1: Figure 1.

**TABLE 3:** Weighted mortality rates by Comprehensive Health Assessment Score (CHAS) groups.

Outcome	CHAS Group	Number of Events	Person‐Years	Rate (per 1000 Person Year)	95% CI
All‐cause mortality	Low (0–4)	618	7509	82.3	75.9–88.7
	Moderate (5–8)	723	14 432	50.1	46.3–53.9
	High (9–12)	201	7535	26.8	23.5–30.1
Cardiac mortality	Low (0–4)	241	7509	32.1	28.2–36.0
	Moderate (5–8)	279	14 432	19.4	17.1–21.7
	High (9–12)	67	7535	9.7	7.9–11.5

*Note:* Rates were weighted for the complex sampling design of NHANES.

Abbreviations: CHAS, Comprehensive Health Assessment Score; CI, confidence interval.

### 3.3 Association Between CHAS and Mortality

**FIGURE 2:**
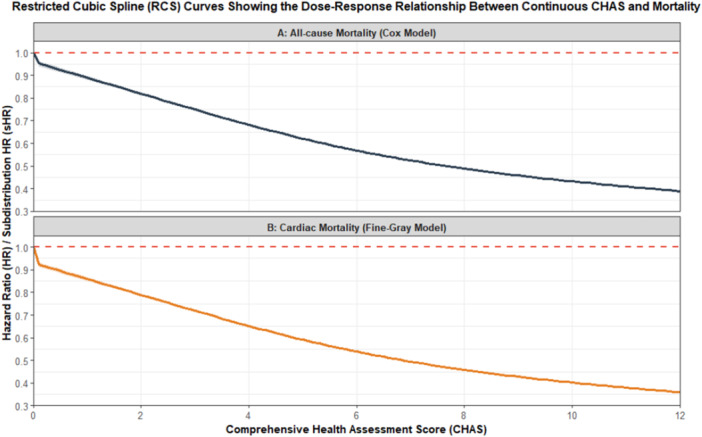
Restricted cubic spline (RCS) curves showing the dose‐response relationship between continuous CHAS and mortality. (A) All‐cause mortality (Cox model); (B) Cardiac mortality (Fine–Gray model). Dashed lines represent 95% CIs. The models were fully adjusted for age, sex, demographics, CHD severity, and survey design variables.

For all‐cause mortality, Kaplan–Meier curves demonstrated a clear gradient of lower mortality with a higher CHAS (log‐rank test, *p* < 0.001; Figure 1). In the Cox proportional hazards models (Table 4), after full adjustment (Model 3), compared with a low CHAS, a moderate CHAS was associated with a 32% lower all‐cause mortality risk (HR = 0.68; 95% CI: 0.59–0.78), and a high CHAS was associated with a 55% lower risk (HR = 0.45; 95% CI: 0.38–0.53). A significant dose–response relationship was observed (P for trend < 0.001). Restricted cubic splines confirmed a linear inverse association between continuous CHAS score and all‐cause mortality (HR per 1‐point increase in CHAS score = 0.87; 95% CI: 0.85–0.89; Figure 2).

**TABLE 4:** Hazard ratios (HRs) and 95% confidence intervals (CIs) for the association between CHAS and mortality.

Outcome	CHAS Group	Model 1 (Age + Sex)	Model 2 (Model 1 + Demographics + CHD Severity)	Model 3 (Full Adjustment)	P for Trend
All‐cause mortality	Low (0–4)	Reference	Reference	Reference	< 0.001
	Moderate (5–8)	0.57 (0.50–0.65)	0.61 (0.53–0.70)	0.68 (0.59–0.78)	
	High (9–12)	0.32 (0.27–0.38)	0.38 (0.32–0.45)	0.45 (0.38–0.53)	
Cardiac mortality	Low (0–4)	Reference	Reference	Reference	< 0.001
	Moderate (5–8)	0.51 (0.42–0.62)	0.56 (0.46–0.68)	0.62 (0.51–0.76)	
	High (9–12)	0.25 (0.19–0.33)	0.31 (0.24–0.40)	0.39 (0.30–0.51)	

*Note*: Full adjustment (Model 3) includes Model 2 variables + survey design variables (sampling weight, strata, PSU). All models accounted for the complex sampling design of NHANES. P for trend was calculated using continuous CHAS as a predictor.

Abbreviations: CHAS = Comprehensive Health Assessment Score, CI = confidence interval, HR = hazard ratio (Cox proportional hazards model for all‐cause mortality; Fine–Gray subdistribution HR for cardiac mortality).

**FIGURE 3:**
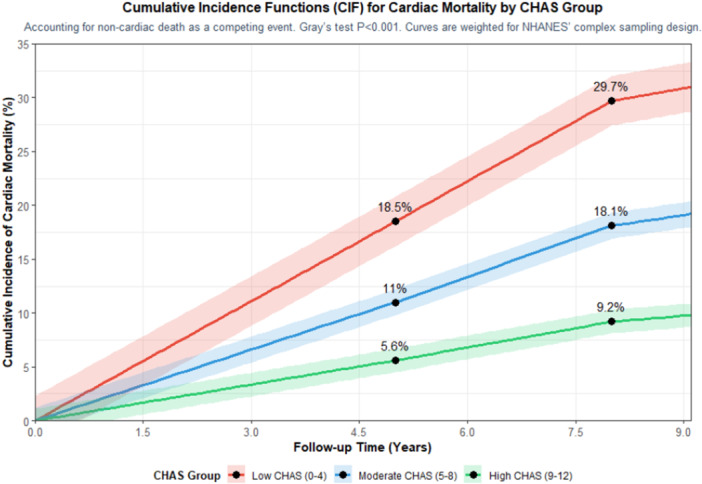
Cumulative incidence functions (CIF) for cardiac mortality by CHAS group (low/moderate/high), accounting for non‐cardiac death as a competing event. Gray's test *p* < 0.001. Curves are weighted for the complex sampling design of NHANES.

For cardiac mortality, cumulative incidence functions (CIFs) demonstrated lower cardiac mortality with a higher CHAS (Gray's test, *p* < 0.001; Figure 3). In the Fine–Gray competing risk models (Table 4), after full adjustment, moderate CHAS was associated with a 38% lower cardiac mortality risk (sHR = 0.62; 95% CI: 0.51–0.76), and high CHAS was associated with a 61% lower risk (sHR = 0.39; 95% CI: 0.30–0.51). Dose–response relationships were also significant (P for trend < 0.001), with a linear inverse association observed in RCS (sHR per 1‐point increase in CHAS = 0.84; 95% CI: 0.81–0.87; Figure 2).

### 3.4 Domain‐Specific Associations

To identify key contributors to the CHAS–mortality association, the independent effects of each CHAS domain (lifestyle, physical function, metabolic health, comorbidity, and medication) on mortality were evaluated while adjusting for all other domains and confounders (Table 5).

**TABLE 5:** Domain‐specific hazard ratios (HRs) for mortality (full adjustment).

Domain	Outcome	Tertile 1 (Low)	Tertile 2 (Moderate)	Tertile 3 (High)	P for Trend
Lifestyle	All‐cause mortality	Reference	0.82 (0.72–0.93)	0.61 (0.53–0.70)	< 0.001
	Cardiac mortality		0.78 (0.65–0.93)	0.57 (0.46–0.71)	< 0.001
Physical Function	All‐cause mortality		0.89 (0.78–1.01)	0.78 (0.68–0.90)	0.002
	Cardiac mortality		0.85 (0.72–1.01)	0.75 (0.62–0.91)	0.003
Metabolic Health	All‐cause mortality		0.86 (0.76–0.98)	0.76 (0.66–0.88)	< 0.001
	Cardiac mortality		0.83 (0.70–0.98)	0.72 (0.59–0.88)	0.001
Comorbidity & Medication	All‐cause mortality		0.79 (0.69–0.91)	0.68 (0.59–0.78)	< 0.001
	Cardiac mortality		0.69 (0.57–0.83)	0.55 (0.44–0.69)	< 0.001

*Note*: Tertiles defined by the domain‐specific score distribution. All models were adjusted for age, sex, demographics, CHD severity, survey design variables, and other CHAS domains.

Abbreviations: CI = confidence interval, HR = hazard ratio (Cox for all‐cause mortality; Fine–Gray for cardiac mortality).

**FIGURE 4:**
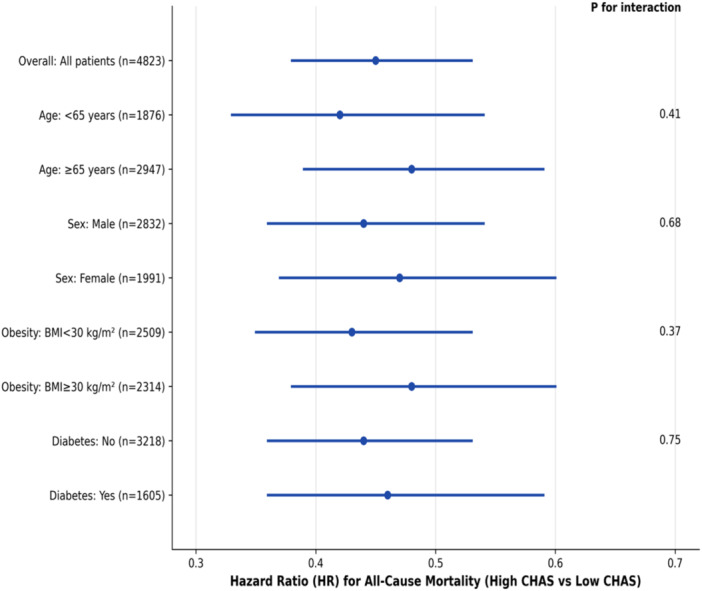
Forest plot of subgroup analyses for the association between high CHAS versus low CHAS and all‐cause mortality. All models were fully adjusted for age, sex, demographics, CHD severity, and survey design variables. Horizontal lines represent 95% CIs; points represent HRs. P for interaction are shown for each subgroup.

In Figure 4, for all‐cause mortality, the lifestyle domain showed the strongest independent association; compared with the lowest tertile, the highest tertile was associated with a 39% lower risk (HR = 0.61; 95% CI: 0.53–0.70). Within this domain, meeting physical activity guidelines (≥ 150 MET‐min/week) was identified as the strongest predictor (HR = 0.71; 95% CI: 0.63–0.80). The comorbidity and medication domain showed the second strongest association (highest vs. lowest tertile: HR = 0.68; 95% CI: 0.59–0.78), driven by the dual use of statins and antiplatelets (HR = 0.58; 95% CI: 0.49–0.69 for cardiac mortality). Because the medication component was intentionally narrow, this domain should be interpreted as reflecting partial rather than exhaustive secondary prevention intensity. The metabolic health and physical function domains also showed significant inverse associations, but with smaller effect sizes (HRs = 0.75–0.78 for highest vs. lowest tertiles).

For cardiac mortality, similar domain‐specific patterns were observed, with the comorbidity and medication domains showing the most pronounced effect (highest vs. lowest tertile: sHR = 0.55; 95% CI: 0.44–0.69).

### 3.5 Subgroup Analyses

Consistent inverse associations between CHAS and mortality were observed across all examined strata, including age (< 65 vs. ≥ 65 years), sex, obesity status (BMI < 30 vs. ≥ 30 kg/m^2^), and diabetes status (P for interaction > 0.05 for all). The association between high versus low CHAS and all‐cause mortality remained robust in both younger (HR, 0.42 [0.33–0.54]) and older participants (HR, 0.48 [0.39–0.59]). These consistent findings suggest that the survival advantage associated with a favorable comprehensive health profile is broadly applicable across demographic and clinical subgroups.

### 3.6 Sensitivity Analyses

All sensitivity analyses confirmed the robustness of the primary findings (Tables 6 and 7). After exclusion of deaths within 12 months of baseline, high CHAS versus low CHAS remained associated with lower all‐cause mortality (HR = 0.47; 95% CI: 0.39–0.56) and cardiac mortality (sHR = 0.41; 95% CI: 0.31–0.54). Results from multiple imputation for missing data were consistent with the complete‐case analysis (all‐cause mortality: HR = 0.46; 95% CI: 0.39–0.55; cardiac mortality: sHR = 0.40; 95% CI: 0.31–0.52). Truncation of extreme CHAS values (1st/99th percentiles) did not materially change the estimates (all‐cause mortality: HR = 0.44; 95% CI: 0.37–0.52; cardiac mortality: sHR = 0.38; 95% CI: 0.29–0.50). Replacement of HEI‐2015 with fruit and vegetable intake (≥ 5 servings/day) as an alternative dietary measure did not alter the association (all‐cause mortality: HR = 0.46; 95% CI: 0.39–0.55). These consistent findings reinforce the stability of the observed associations and minimize concerns regarding reverse causation or measurement bias.

**TABLE 6:** Subgroup analyses for all‐cause mortality (high CHAS vs. low CHAS, full adjustment).

Subgroup	*n*	HR (95% CI)	P for Interaction
Age			0.41
< 65 years	1876	0.42 (0.33–0.54)	
≥ 65 years	2947	0.48 (0.39–0.59)	
Sex			0.68
Male	2832	0.44 (0.36–0.54)	
Female	1991	0.47 (0.37–0.60)	
Obesity			0.37
BMI < 30 kg/m^2^	2509	0.43 (0.35–0.53)	
BMI ≥ 30 kg/m^2^	2314	0.48 (0.38–0.60)	
Diabetes			0.75
No	3218	0.44 (0.36–0.53)	
Yes	1605	0.46 (0.36–0.59)	

*Note*: Full adjustment included age, sex, demographics, CHD severity, and survey design variables.

Abbreviations: BMI = body mass index, CHAS = Comprehensive Health Assessment Score, CI = confidence interval, HR = hazard ratio (Cox proportional hazards model).

**TABLE 7:** Sensitivity analyses for the association between high CHAS versus low CHAS and mortality (full adjustment).

Sensitivity Analysis Scenario	Outcome	HR/sHR (95% CI)
Primary analysis (complete case)	All‐cause mortality	0.45 (0.38–0.53)
	Cardiac mortality	0.39 (0.30–0.51)
Exclude deaths within 12 months	All‐cause mortality	0.47 (0.39–0.56)
	Cardiac mortality	0.41 (0.31–0.54)
Multiple imputation (MICE, *n* = 20)	All‐cause mortality	0.46 (0.39–0.55)
	Cardiac mortality	0.40 (0.31–0.52)
Truncate CHAS (1st/99th percentiles)	All‐cause mortality	0.44 (0.37–0.52)
	Cardiac mortality	0.38 (0.29–0.50)
Alternative diet measure (fruit/vegetable intake)	All‐cause mortality	0.46 (0.39–0.55)
	Cardiac mortality	0.40 (0.31–0.52)

*Note*: All models accounted for the complex sampling design of NHANES.

Abbreviations: CHAS = Comprehensive Health Assessment Score, CI = confidence interval, HR = hazard ratio (Cox model), MICE = multiple imputation by chained equations, sHR = subdistribution hazard ratio (Fine–Gray model).

## 4 Discussion

In this prospective cohort study of 4823 U.S. adults with CHD followed for a median of 6.2 years, three key findings were observed. First, a graded and robust inverse association between the study‐derived CHAS and mortality was identified. Participants with a high CHAS (9–12 points) had a 55% lower risk of all‐cause mortality and a 61% lower risk of cardiac mortality compared with those with a low CHAS (0–4 points), even after full adjustment for confounders and the complex sampling design of NHANES. The relationship was linear across the entire CHAS range, confirming a clear dose–response association between multidimensional health status and survival. Second, domain‐specific analyses identified that lifestyle factors—particularly meeting physical activity recommendations (≥ 150 MET‐min/week)—and comorbidity & medication management, primarily the concurrent use of statins and antiplatelets, were the strongest independent predictors of survival. These results reinforce the synergistic benefits of combining lifestyle optimization with evidence‐based pharmacotherapy. Third, the CHAS–mortality association was found to be consistent across subgroups defined by age, sex, obesity status, and diabetes, indicating that the multidimensional health assessment framework is broadly applicable to diverse CHD populations. CHAS should therefore be regarded as a research‐oriented summary framework rather than an externally validated clinical tool.

These findings extend the original research focus on exercise habits and CHD mortality by advancing understanding in three ways. First, the analysis moves beyond single‐factor approaches to demonstrate that the joint optimization of lifestyle, physical function, metabolic health, and comorbidity management is associated with greater mortality reduction than any individual component. While prior studies have shown that physical activity alone reduces CHD mortality risk by 20%–30% [8], the present findings indicate that combining physical activity with pharmacologic and metabolic optimization is associated with substantially lower cardiac mortality, highlighting the additive value of an integrated approach. Second, the use of nationally representative NHANES data enhances generalizability. The observed mortality rates are consistent with national CHD registry data, suggesting that the findings reflect real‐world outcomes [9, 10]. This supports the original protocol's rationale that NHANES represents an “ideal data source” for causal inference in diverse populations. The interpretation is also aligned with broader composite cardiovascular health metrics that predict hard outcomes in U.S. adults, although CHAS itself has not yet undergone external validation [11]. Third, the CHAS classification provides clinically actionable thresholds that correspond directly to established secondary prevention guidelines, offering a quantifiable tool for patient stratification and risk communication.

These observed associations are likely attributable to overlapping biological mechanisms. Endothelial function is improved, systemic inflammation is reduced, and cardiopulmonary fitness is enhanced through regular physical activity, thereby slowing the progression of atherosclerosis. These effects are complemented by pharmacologic management with statins and antiplatelets through plaque stabilization and thrombosis prevention. Grip strength may capture physiological reserve and frailty‐related vulnerability, while lower CRP levels may reflect reduced residual inflammatory risk [12]. Furthermore, vascular stress and myocardial workload are reduced through optimal metabolic control, as reflected in blood pressure, glycemia, and lipid levels. The linear dose–response relationship between CHAS and mortality suggests that even modest improvements across these domains may yield meaningful survival benefits, supporting a “treat‐to‐target” strategy for secondary prevention of CHD.

This study has several limitations. Exposure variables such as physical activity and CHD diagnosis were self‐reported, introducing potential misclassification. However, validated NHANES instruments were used, strict data‐quality exclusions were applied, and robustness was confirmed across multiple sensitivity analyses. Despite comprehensive adjustment for confounders, unmeasured factors (e.g., genetic predisposition or cardiac rehabilitation adherence) may remain unmeasured. Reverse causation is also possible, although the exclusion of deaths within the first year of follow‐up did not materially change the results. In addition, CHAS was constructed as an unweighted composite, which improves transparency but assumes comparable contributions from heterogeneous components. The medication component was limited to statins and antiplatelet agents, and the comorbidity component was limited to diabetes, CKD, and COPD; accordingly, the score may not fully capture pharmacologic management or the total disease burden. The primary analysis was also based on complete cases; therefore, missing dietary and functional measurements may have introduced selection bias despite the consistency of the MICE results. The conceptual overlap between domains cannot be fully excluded. Finally, the follow‐up ended in 2019, precluding the evaluation of longer‐term outcomes, although the median follow‐up of 6.2 years captured a substantial number of mortality events.

These findings have important clinical implications. Multidimensional risk stratification can be enabled by the CHAS, allowing the identification of high‐risk CHD patients who may benefit from intensified interventions, such as structured exercise programs or medication adherence support. Specific CHAS components provide concrete, measurable goals, such as achieving ≥ 150 MET‐min/week of physical activity or maintaining LDL cholesterol < 70 mg/dL, that can be directly integrated into clinical practice. At the population level, these findings support the further evaluation of CHAS‐informed secondary prevention strategies rather than immediate routine implementation.

Future research should extend these findings by incorporating longer follow‐ups as additional mortality data become available, conducting interventional studies to test CHAS‐guided strategies, and refining the CHAS framework with emerging biomarkers and digital health metrics. Comparisons between the weighted and unweighted versions of the CHAS, explicit reporting of component‐level missingness, and external validation in independent CHD cohorts will be particularly important.

## 5 Conclusions

A higher CHAS, which reflects a favorable lifestyle, functional, metabolic, and comorbidity profiles, as well as a lower inflammatory burden, is strongly associated with reduced cardiac and all‐cause mortality among U.S. adults with CHD. These results highlight the value of a multidimensional approach to secondary prevention, and the importance of integrating lifestyle modifications with optimal pharmacologic and metabolic control is highlighted. Although the CHAS may be considered a pragmatic research framework, further methodological refinement and external validation are required before routine clinical adoption.

## Author Contributions

Yanqiong Li and Lixia Yang contributed equally to this work. Yanqiong Li: Conceptualization, methodology, investigation, data analysis, writing – original draft. Lixia Yang: Investigation (co‐equal), data curation, validation. Jiaoyang Lu: Formal analysis, investigation support. Yanhui Wang: Computational modeling, statistical analysis. Ling Cheng: Literature review, data interpretation assistance. Zhou Zuo: Experimental validation, figure preparation. Zhenlin Fu: Resources, project administration. Peng Chen: Supervision, funding acquisition, writing – review and editing, corresponding author.

## Conflicts of Interest

The authors declare no conflicts of interest.

## Supporting information

Supporting File

## Data Availability

The data that support the findings of this study are available from the corresponding author upon reasonable request. The NHANES datasets and linked mortality files analyzed in this study are publicly available from the CDC/NCHS website: https://wwwn.cdc.gov/nchs/nhanes/default.aspx.

## References

[1] E. J. Benjamin , P. Muntner , A. Alonso , et al., “Heart Disease and Stroke Statistics‐2019 Update: A Report From the American Heart Association,” Circulation 139 (2019): e56–e528, 10.1161/CIR.0000000000000659. 30700139

[2] P. CfDCa . NHANES 1999–2018 Survey Documentation and Codebooks. In: Atlanta: CDC/NCHS; 2020.

[3] National Center for Health Statistics . The National Health and Nutrition Examination Survey, accessed May 9, 2024, https://www.cdc.gov/nchs/nhanes/index.htm.

[4] World Health Organization . International Classification of Diseases, 10th Revision (ICD‐10). Geneva: WHO; 2019.

[5] M. D. Jensen , D. H. Ryan , C. M. Apovian , et al., “2013 AHA/ACC/TOS Guideline for the Management of Overweight and Obesity in Adults: A Report of the American College of Cardiology/American Heart Association Task Force on Practice Guidelines and The Obesity Society,” Circulation 129 (2014): S102–S138, 10.1161/01.cir.0000437739.71477.ee. 24222017 PMC5819889

[6] S. M. Grundy , N. J. Stone , A. L. Bailey , et al., “2018 AHA/ACC/AACVPR/AAPA/ABC/ACPM/ADA/AGS/APhA/ASPC/NLA/PCNA Guideline on the Management of Blood Cholesterol: A Report of the American College of Cardiology/American Heart Association Task Force on Clinical Practice Guidelines,” Circulation 139 (2019): 1082, 10.1161/CIR.0000000000000625. PMC7403606 30586774

[7] P. M. Ridker , N. Rifai , L. Rose , J. E. Buring , and N. R. Cook , “Comparison of C‐Reactive Protein and Low‐Density Lipoprotein Cholesterol Levels in the Prediction of First Cardiovascular Events,” New England Journal of Medicine 347 (2002): 1557–1565. 12432042 10.1056/NEJMoa021993

[8] J. Sattelmair , J. Pertman , E. L. Ding , H. W. Kohl , W. Haskell , and I.‐M. Lee , “Dose Response Between Physical Activity and Risk of Coronary Heart Disease: A Meta‐Analysis,” Circulation 124 (2011): 789–795. 21810663 10.1161/CIRCULATIONAHA.110.010710 PMC3158733

[9] G. T. O'Connor , J. E. Buring , S. Yusuf , et al., “An Overview of Randomized Trials of Rehabilitation With Exercise After Myocardial Infarction,” Circulation 80 (1989): 234–244, 10.1161/01.cir.80.2.234. 2665973

[10] V. L. Roger , A. S. Go , D. M. Lloyd‐Jones , et al., “Heart Disease and Stroke Statistics‐‐2012 Update: A Report From the American Heart Association,” Circulation 125 (2012): 2, 10.1161/CIR.0b013e31823ac046. PMC4440543 22179539

[11] E. S. Ford , K. J. Greenlund , and Y. Hong , “Ideal Cardiovascular Health and Mortality From All Causes and Diseases of the Circulatory System Among Adults in the United States,” Circulation 125, no. 8 (2012): 987–995, 10.1161/CIRCULATIONAHA.111.049122. 22291126 PMC4556343

[12] B. Larcher , D. Zanolin‐Purin , A. Vonbank , et al., “Usefulness of Handgrip Strength to Predict Mortality in Patients With Coronary Artery Disease,” American Journal of Cardiology 129 (2020): 5–9. 32580913 10.1016/j.amjcard.2020.05.006

