# Supplementary material for: Association Between Comprehensive Health Assessment and Cardiac Mortality and All‐Cause Mortality in Patients With Coronary Heart Disease
Source: Clin Cardiol. 2026 Jun 15;49(6):e70378. doi: 10.1002/clc.70378 (PMC13267986; doi:10.1002/clc.70378)
Supplement: Supplementary file 1 — Supporting File [file CLC-49-e70378-s001.docx]

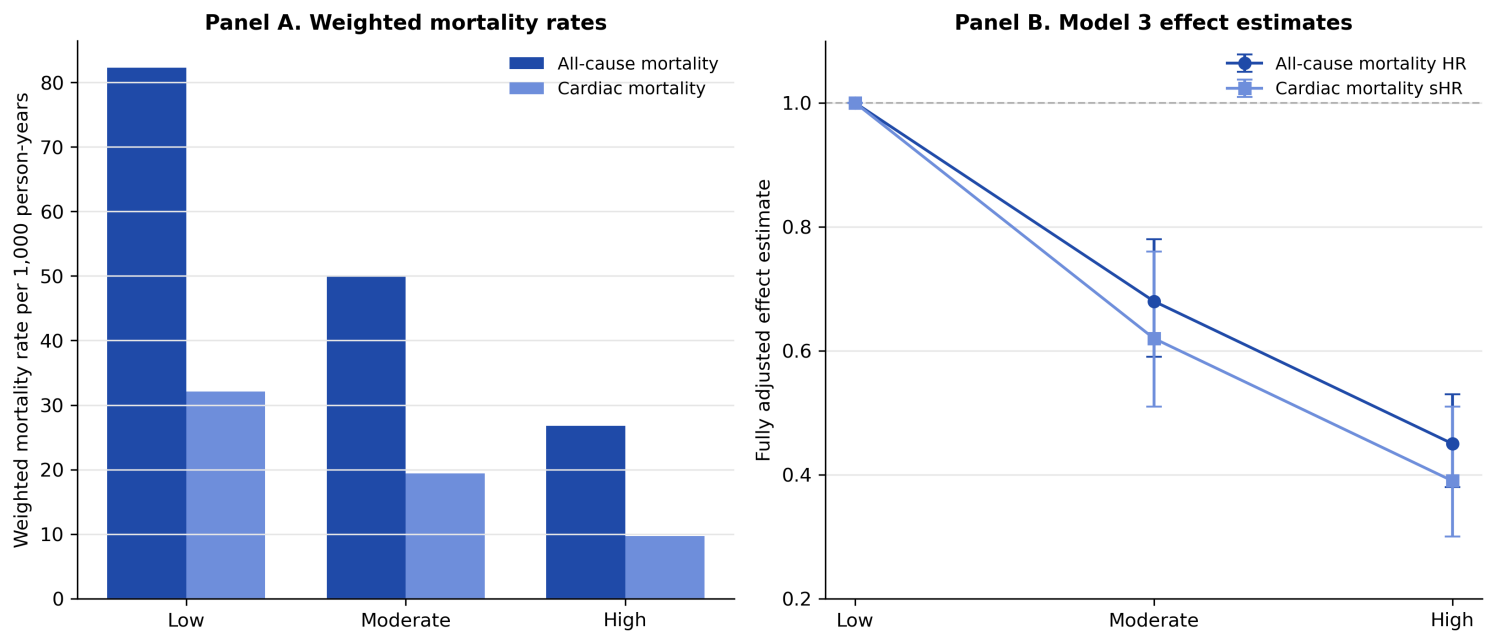


**Supplementary Figure S1.** Weighted mortality rates and fully adjusted effect estimates for the association between CHAS categories and mortality outcomes.

Panel A shows the weighted mortality rates per 1,000 person-years; Panel B shows the fully adjusted HRs/sHRs from Model 3. The figure has been improved to enhance visual readability, as suggested by the reviewer.
